# Elucidating postoperative dynamics in tractional retinal detachment: a systematic review and meta-analysis of structural and functional outcomes following diabetic vitrectomy, including an analysis of postoperative complications

**DOI:** 10.1186/s12886-024-03820-z

**Published:** 2024-12-24

**Authors:** Miguel A. Quiroz-Reyes, Erick A. Quiroz-Gonzalez, Miguel A. Quiroz-Gonzalez, Virgilio Lima-Gomez

**Affiliations:** 1 The Retina Department of Oftalmologia Integral ABC (Medical and Surgical Nonprofit Organization), Affiliated with the Postgraduate Studies Division at the National Autonomous University of Mexico, Lomas de Chapultepec, Av. Paseo de las Palmas 735 Suite 303, Lomas de Chapultepec, Mexico City, 11000 Mexico; 2 Juarez Hospital, Public Assistance Institution (Nonprofit Organization), Av. Politecnico Nacional 5160, Colonia Magdalena de las Salinas, Mexico City, 07760 Mexico

**Keywords:** Diabetic retinopathy, Diabetic tractional retinal detachment, Postoperative structural outcomes, Perfusion markers, Pars plana vitrectomy, Swept-source optical coherence tomography angiography, Vessel density, Diabetic vitrectomy complications, Choroidal vascularity index, Choriocapillaris flow area

## Abstract

This meta-analysis aims to evaluate the efficacy and safety of diabetic vitrectomy for treating tractional retinal detachment (TRD) by assessing visual acuity improvements, retinal reattachment success rates, and the frequency of postoperative complications. A comprehensive literature search identified 1,211 studies, 30 of which met the inclusion criteria and were analyzed. The meta-analyses were conducted via random effects models to account for heterogeneity. The primary outcomes were visual acuity improvement and retinal reattachment success, whereas postoperative complications were analyzed as secondary outcomes. Funnel plots, trim-and-fill methods, and Egger’s regression tests were employed to assess and adjust for publication bias. The meta-analysis included data from 1,844 eyes across 30 studies. The pooled mean difference (MD) in visual acuity following diabetic vitrectomy was 0.80 logMAR (95% CI: 0.58–1.01, *p* < 0.0001), with significant heterogeneity (I² = 95%). The trim-and-fill method adjusted the MD to 1.15 (95% CI: 0.85–1.45), indicating potential publication bias. The pooled proportion of retinal reattachment success was 94.63% (95% CI: 90.88–96.89%), with substantial heterogeneity (I² = 82.2%). Egger’s regression test confirmed publication bias in the retinal reattachment data (t = 6.07, df = 25, *p* < 0.0001). The most common postoperative complications were vitreous hemorrhage (8.2%, 0–41%), elevated intraocular pressure (7.3%, 0–25%), and cataract formation (10.3%, 0–43%). Diabetic vitrectomy significantly improves visual acuity and achieves high retinal reattachment success rates in patients with TRD despite considerable heterogeneity across studies. However, a critical limitation of the current literature is the lack of comprehensive reporting of essential markers of perfusion, such as the choroidal vascularity index and choriocapillaris flow area, which are crucial for a thorough understanding of the impact of vitrectomy on ocular perfusion and its correlation with structural and functional outcomes. These findings also highlight the need for vigilant postoperative management to mitigate common complications. Adjustments for publication bias via the trim-and-fill method suggest that the initial estimates of efficacy may be optimistic, but the overall benefits of the procedure remain substantial. Further research is warranted to standardize surgical techniques, include detailed perfusion outcomes, and enhance the reliability of future meta-analyses. Trial registration: Retrospectively registered.

**Supplementary Information:**

The online version contains supplementary material available at 10.1186/s12886-024-03820-z.

## Introduction

Diabetes is a significant health concern, affecting over 460 million patients globally, with a projected rise in the coming decades [1]. One major complication of diabetes is proliferative diabetic retinopathy (PDR), which affects around 93 million people worldwide, with one-quarter experiencing severe vision loss [2]. In PDR, fibrovascular membrane formation and contraction adhering to the retina result in tractional retinal detachment (TRD), significantly contributing to visual loss in diabetic retinopathy (DR) [3, 4]. In hyperglycemia, the secretion of angiogenic factors, such as vascular endothelial growth factor (VEGF), leads to developing fibrovascular membranes [3]. Despite the implementation of diabetic eye screening initiatives, improvements in diabetic management [5], and treatments for PDR, many individuals with diabetes continue to experience severe complications, such as TRD [6].

In TRD, when the fovea is affected, early pars plana vitrectomy (PPV) is recommended to prevent irreversible visual loss. However, TRD without macular involvement should be carefully monitored since it can potentially remain stable [7]. TRD is the most common indication for eye vitrectomy with PDR [4]. Approximately 5% of patients with PDR require PPV for TRD despite being treated with panretinal photocoagulation (PRP) [6]. In the last 25 years, there have been advances in surgical techniques, including small-gauge vitrectomy and new tamponade agents; anti-VEGF medications are also used preoperatively and intraoperatively [8–10]. TRD surgical repair is one of the most technically challenging ocular surgeries due to the fragility of the ischemic retina and the complexity of extensive fibrovascular membranes. Retinal ischemia assessment is typically performed via fundus fluorescein angiography (FFA). However, this approach has several limitations, as it is time-consuming and exposes patients to potential systemic adverse effects [11].

Moreover, it necessitates intravenous access, which can be difficult for patients with severe diabetes. It also provides limited detailed characterization of the retinal microvasculature surrounding neovascularization. Additionally, identifying the margins, vascularity, and elevation of fibrovascular membranes on FFA is challenging because of hyperfluorescence and potential leakage in traction areas, even without neovascularization [12]. These factors restrict the application of FFA in clinical practice.

Optical coherence tomography (OCT) has emerged as an essential tool for diagnosing and treating various retinal diseases. It offers a noninvasive and reproducible method, generating cross-sectional, three-dimensional retina images [13]. Moreover, OCT can identify specific regions of the PVD, which are frequently confined and partial in TRDs. These identified areas can be utilized as critical points for surgical interventions, facilitating the advancement of a complete PVD and aiding in the separation or division of fibrovascular plaques [14]. Nonetheless, structural OCT does not provide information on retinal ischemia or the vascularity of preretinal membranes [12]. OCT angiography (OCTA) is an advancement over OCT, offering enhanced resolution details on retinal and choroidal blood flow to deliver more precise microcirculation information across various levels. Despite its improvements, traditional OCTA has a restricted field of view (FOV) for the retina, and imaging quality diminishes as the scanned region expands. In recent years, swept-source OCTA (SS-OCTA), a third-generation spectral domain OCT (SD-OCT), has been progressively applied in diagnosing, treating, and prognosis of TRD after several improvements [15]. The structural OCT transition to SS-OCTA represents a crucial shift in understanding and managing TRDs by providing comprehensive insights into anatomical and microcirculatory aspects, offering a potential approach for refined diagnostic and treatment strategies.

This study aims to elucidate, describe, and compare postoperative structural (SD-OCT, SS-OCT) and postoperative perfusion outcomes (OCTA), such as vessel density (VD) quantification in the superficial and deep capillary plexuses of the retina and choroidal perfusion markers, such as the choroidal vascularity index (CVI) and choriocapillaris flow area (CFA) in postoperative eyes suffering from TRD at 4, 6, 12, or more months, by performing a systematic review and meta-analysis of the literature to evaluate anatomic and functional outcomes, providing insights into the factors influencing surgical success.

## Methodology

### Literature search

This systematic review and meta-analysis followed the Preferred Reporting Items for Systematic Reviews and Meta-analyses (PRISMA) reporting guidelines [16]. A detailed and thorough literature search was conducted in the Web of Science, MEDLINE, and Embase databases for potentially eligible studies using specific keywords, including “diabetic tractional retinal detachment,” “vitrectomy,” “visual acuity,” “retinal reattachment,” “choroidal vascularity index,” “choriocapillaris flow area,” “postoperative complications,” and “ocular perfusion.” Eligible reference lists were also scrutinized to identify additional studies. The final literature search was conducted on August 1, 2024, and no time frame limits were placed on the literature search.

### Study selection criteria

Two reviewers (MAQR and EAQG) independently reviewed all the retrieved studies from the databases. Studies were classified as potentially eligible based on titles and abstracts. Potentially eligible studies were further subjected to full-text screenings to select studies perfectly aligned with our objectives. They fulfilled the eligibility criteria of the systematic review and meta-analysis. The retrieved studies were considered eligible based on the following inclusion criteria: (a) randomized clinical trials (RCTs), case-control studies, and prospective or retrospective study designs; (b) participants: studies presenting outcomes of PPV for TRD; and (C) studies having a minimum follow-up duration of 3 months for enrolled patients. Any discrepancies were resolved by discussion between reviewers or with the intervention of a conciliator. The supplementary information file contains details on the search strategy and prespecified participants, interventions, comparisons, and outcomes (PICO) framework.

### Data extraction

Data extraction was conducted by a single reviewer (MAQR) and subsequently validated by a second reviewer (MAQG) to ensure precision. The investigation focused on specific data points, including but not limited to eyes with TRD at 4, 6, 12, or more months after a successful operation. Their retinal and choroidal perfusion markers were correlated with the final best-corrected visual acuity (BCVA), and 12 months (± 3 months) and additional time points postoperatively were considered if available. The extracted information included the following: the count (percentage) of individuals’ eyes with a flat retina following singular surgery and those with a flat retina after undergoing multiple surgeries; BCVA; the proportion of individuals (eyes) achieving a visual acuity of 0.30 logMAR (approximately Snellen equivalent, 6/12) or better; and 1.0 logMAR (approximately Snellen equivalent, 6/60) or worse, as well as intraoperative and postoperative complications. Data on OCT and OCTA outcomes, such as CVI, CFA, and other perfusion parameters, were extracted from the included studies when reported. Quantitative data were recorded as means and standard deviations (when available), while qualitative findings, such as narrative descriptions of OCT findings, were synthesized descriptively. Due to variability in reporting formats and outcome definitions, a standardized framework for interpreting OCTA metrics was used when feasible. Studies lacking sufficient details on OCT or OCTA data were excluded from perfusion-specific analyses.

Demographic data, precisely age and sex, were sourced from eligible studies, whereas information on other demographic characteristics, such as race and ethnicity, was not extracted because of infrequent reporting. Additionally, data related to preoperative patient characteristics, surgical procedures during PPV, preoperative or intraoperative anti-VEGF injections, and the number of surgeries required for retinal reattachment were also documented when available. The number of studies included in each analysis varied depending on the availability and quality of reported data. For instance, while most studies reported visual acuity and anatomical success outcomes, fewer studies included detailed data on postoperative complications or perfusion metrics, such as CVI. To ensure transparency, the exact number of studies included in each analysis is specified in the Results section, and studies with incomplete or inconsistent data were excluded from specific analyses.

### Risk of bias and quality assessment

Potential bias was assessed via the National Institutes for Health quality assessment tools pertinent to the specific study design. Two independent reviewers (MAQR and VLG) performed the evaluation, and any disparities were resolved through discussion or the involvement of a third reviewer. The National Institutes for Health quality assessment tools categorize studies as either “good,” indicating a low risk of bias; “fair,” signifying some risk of bias that is not sufficient to compromise the study’s validity; or “poor,” indicating a notable risk of bias.

### Meta-analysis

For the meta-analyses of proportions, we utilized the meta prop function from the meta package in R (version 4.3.1). A random-intercept logistic regression model was employed, following the specifications outlined by Schwarzer et al. [17]. The maximum likelihood estimator for τ^2, a logit transformation of proportions, and a Clopper–Pearson confidence interval for individual studies were applied. Continuous variables, such as the mean final BCVA, were pooled via an inverse variance method with restricted maximum likelihood estimation of random effects [18]. Study heterogeneity was evaluated visually by examining the intersection of confidence intervals and I2 values across different studies—planned heterogeneity investigation involved adding study-level categorical covariates to the model. Continuous study-level variables (e.g., mean age) were dichotomized using median values and included as covariates. Preoperative or intraoperative anti-VEGF medications were dichotomized as studies with 100% and less than 100%.

In contrast, the gauge was dichotomized into studies using exclusively standard 20-gauge vitrectomy and exclusively small-gauge microincision vitrectomy surgery (MIVS) systems (23, 25, and 27 gauge). A post hoc analysis compared outcomes from studies published during or after 2016 with those published before 2016, allowing a comparison of recent and older studies. All relevant study arms were included in the case of data from RCTs. Postoperative complications are presented via simple descriptive statistics.

## Results

**Fig. 1:**
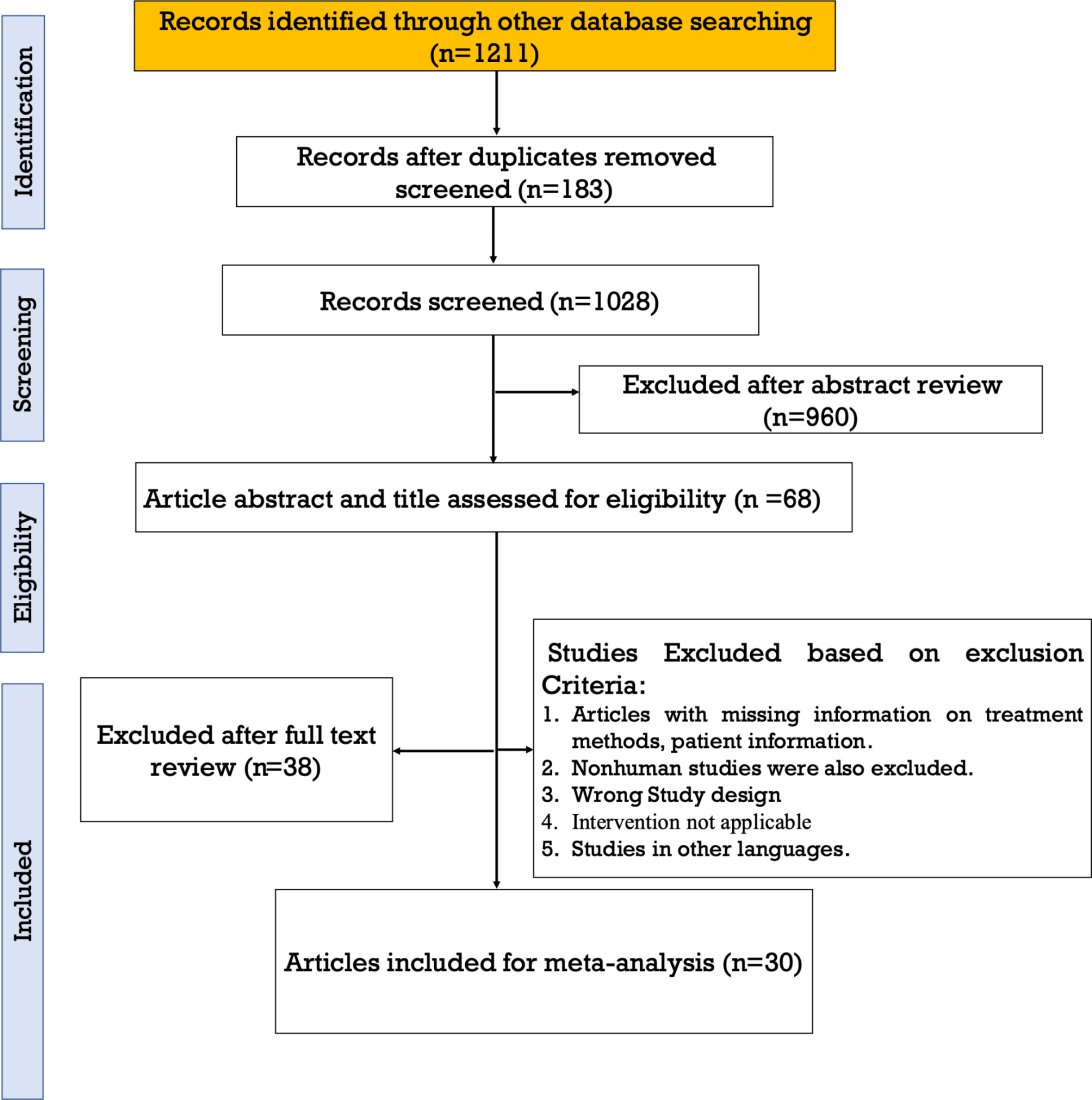
PRISMA flow chart showing the detailed literature screening process

### Study selection and demographic data

In total, 1211 studies were identified through database searches, with 183 duplicates removed, leaving 1028 records for screening. Following an abstract review, 960 studies were excluded, and 68 articles underwent full-text assessment. Of these, 38 studies were excluded based on specific criteria, such as missing treatment methods or patient information, nonhuman studies, incorrect study designs, nonapplicable interventions, or non-English languages. Ultimately, 30 studies were included in the meta-analysis. The PRISMA flowchart details the selection process (see Fig. 1).

The included studies provided comprehensive demographic data, encompassing 1844 eyes from diverse patient cohorts. The mean age of the patients ranged from 42 to 55.3 years across the studies. The sex distribution was reported in several studies, comprising 511 males and 456 females. For example, Rahimy et al. (2015) [19] included 62 eyes from 58 patients (35 males, 23 females) with a mean age of 48.1 years and an HbA1c of 8.4%. Steinmetz et al. [20] included 67 eyes from 62 patients aged 46 years on average, 37% of whom had type 1 diabetes. Velez-Montoya et al. [21] included 12 patients (5 males, seven females) with a mean age of 52.25 years. Preoperative visual acuity varies, with studies reporting values ranging from 1.17 to 2.22 logMAR. Previous treatments, such as PRP and anti-VEGF injections, were commonly noted, reflecting the varied treatment histories of the patients. Comorbidities such as hypertension and nephropathy have also been reported in several studies, highlighting the complexity of patient populations. The detailed demographic and anatomic outcome data are presented in Tables 1 and 2, respectively.

**Table 1:** Demographic data

Study	Sample Size	Age (years, mean ± SD)	Gender (M/F)	Duration of Diabetes (years)	HbA1c (%)	Preoperative Visual Acuity (logMAR)	Type of Diabetes (Type 1/Type 2)	Comorbidities	Previous Treatments
Rahimy et al., 2015 [19]	62 eyes of 58 patients	48.1 ± 9.4	35/23	Not specified	8.4 ± 1.9	2.0 ± 0.5	Not specified	Not specified	54.8% PRP, 12.9% bevacizumab
Steinmetz et al., 2002 [20]	67 eyes of 62 patients	46 (range 25–74)	30/32	Not specified	Not specified	Not specified (Functional vision described)	23 (37%) Type 1, 39 (63%) Type 2	Not specified	93% prior PRP
Dikopf et al., 2015 [8]	70 eyes of 55 patients	47.7 (range 23–76)	23/32	Not specified	Not specified	1.59 (20/800, SD 0.88)	Not specified	Not specified	Not specified
Zghal et al., 2019 [22]	52 eyes	50.62	Not specified	Not specified	9.3	1.80	Not specified	Not specified	Not specified
Russell et al., 2021 [12]	31 eyes of 21 patients	49.3 (SD 9.92)	Not specified	Not specified	8.8 (SD 2.4)	Not specified in logMAR, Snellen described	76% Type 2	Not specified	Not specified
Velez-Montoya et al., 2011 [21]	12 patients	52.25 ± 8.6	5/7	Not specified	Not specified	Varied (BCVA in Snellen)	Not specified	Not specified	None received intravitreal bevacizumab preoperatively
Mikhail et al., 2016 [9]	109 eyes of 73 patients	53.9 ± 9.2	39/34	17.4 ± 11.2	Not specified	1.17 (20/300)	41 (56%) Type 1, 32 (44%) Type 2	Not specified	50% received anti-VEGF
Heimann et al., 1989 [23]	106 eyes of 91 patients	Median 50 (range 19–77)	Not specified	Not specified	Not specified	Varied (reported in categories)	67 (74%) Type 1, 24 (26%) Type 2	28 had iris neovascularization	Approximately 50% had laser coagulation, 7 had cryoapplication, 31 had previous vitrectomy
Tao et al., 2010 [24]	168 eyes of 150 patients	52 ± 11.6 (range 20–82)	73 women	Insulin dependent: 13.0 ± 7.8 (range 8–36), oral medication: 11.8 ± 6.0 (range 0.25-30)	Not specified	2.22 ± 1.22	21 (14%) insulin dependent, 129 (86%) oral medication	6 eyes (4%) had iris neovascularization, 49 eyes (29%) had severe VH, 47 eyes (28%) had macular TRD	81 eyes (48%) had PRP, 33 eyes (20%) had combined cataract surgery and intraocular lens implantation
Kumar et al., 2014 [25]	50 eyes of 50 patients	23-G: 58.40 ± 5.55, 25-G: 61.86 ± 4.96	23-G: 16/9, 25-G: 14/11	Not specified	Not specified	23-G: 1.28 ± 0.36, 25-G: 1.29 ± 0.42	Type 2 diabetes mellitus	None specified	All eyes had previous PRP
Gurelik et al., 2024 [26]	25 eyes of 25 patients	Not specified	Not specified	Not specified	Not specified	Group 1: 1.90 ± 0.43, Group 2: 1.93 ± 0.41	Not specified	Not specified	Not specified
Arevalo et al., 2014 [27]	114 eyes of 114 patients	45 (range 21–85)	Not specified	Not specified	Not specified	Not specified (presented as ETDRS lines)	Not specified	Not specified	Not specified
Stoffelns and Dick, 2000 [28]	84 eyes	Not specified	Not specified	Not specified	HbA1c < 9.3 or ≥ 9.3	Not specified	Type I and Type II diabetes	Not specified	Not specified
Qamar, Saleem, and Saleem, 2013 [29]	84 eyes	Mean age: 52 (40–60)	35/40	Insulin dependent: 14 (20–36); Noninsulin dependent: 11 (10–30)	Not specified	2.00 ± 1.24	Insulin dependent: 14.7%, Noninsulin dependent: 85.3%	Pseudophakic: 6.0%, Cataract: 39.2%, Clear lenses: 54.7%	PRP performed in 47.6% of eyes; Severe VH: 29.7%; Macular detachment: 42.8%
Awan et al., 2023 [30]	196 eyes	55.3 ± 11.3	83/93	Not specified	Not specified	1.86 ± 0.59	Not specified	Not specified	Not specified
Abunajma et al., 2016 [31]	96 eyes	54.56 ± 12.8	65/23	Not specified	Not specified	Not specified	Type 1: 18, Type 2: 78	Hypertension, Nephropathy	Previous retinal laser procedures, intravitreal bevacizumab
Wang et al., 2016 [32]	66 eyes of 58 patients	Four-port: 53.1 ± 10.1, Conventional: 51.2 ± 10.6	22/36	Not specified	Not specified	Four-port: 2.04 ± 0.76, Conventional: 2.21 ± 0.67	Type 1: 27, Type 2: 31	Hypertension, NVG	Previous PRP, intravitreal injections excluded
Jain et al., 2019 [33]	33 eyes	Not specified	Not specified	Not specified	Not specified	1.80	Not specified	Not specified	15 patients received preoperative intravitreal ranibizumab
Sokol et al., 2018 [34]	90 eyes of 79 patients	Not specified	Not specified	Not specified	Not specified	Not specified	Not specified	Not specified	Not specified
Sokol et al., 2019 [35]	69 eyes of 61 patients	47 (21–65)	36/25	Not specified	9%	1.84 ± 0.61	Not specified	Not specified	Not specified
Falavarjani et al., 2022 [36]	38 eyes	54.2 ± 9.4	Not specified	Not specified	Not specified	Not specified	Not specified	Not specified	Not specified
Duong et al., 2023 [37]	79 eyes	44.8 ± 12.0	35/44	Not specified	8.9 ± 2.2	1.57 ± 0.72	Type 1: 31 (39.2%), Type 2: 48 (60.8%)	Hypertension (79.7%), severe peripheral vascular disease (17.7%)	Not specified
Meier and Wiedemann, 1997 [38]	28 eyes	55 (22–71)	10/18	Type 1: 23.6 (16–32), Type 2: 11.2 (5–33)	Not specified	Defective LP to 0.1	Type 1: 13, Type 2: 15	Nephropathy, polyneuropathy, cardiovascular changes	PRP, focal/grid photocoagulation, cataract extraction
Arevalo, 2008 [39]	57 eyes	42 (23–84)	Not specified	Not specified	Not specified	Not specified	Not specified	Not specified	Not specified
Rice et al., 1983 [40]	197 eyes	21–76 (median 42)	93/94	Not specified	Not specified	Not specified	Not specified	Not specified	Not specified
La Heij et al., 2004 [41]	44 eyes	Not specified	19/14	Not specified	Not specified	Median 20/800	Type 1: 13, Type 2: 31	Hypertension, nephropathy, proteinuria, cardiovascular complications, varicose ulcer, neurologic complications, cerebrovascular accidents	PRP, cryocoagulation, macular photocoagulation, lensectomy, cryotherapy, silicone oil, gas tamponade
Moon et al., 2015 [42]	6 eyes of 4 patients	56 (range 43–56)	2/2	Not specified	Not specified	Not specified	Not specified	Not specified	Not specified
Miller et al., 1980 [43]	43 eyes	41 (men), 49 (women)	19/22	Juvenile onset: 25, Adult onset: 17	Not specified	Not specified	Not specified	Not specified	Scatter photocoagulation, focal photocoagulation, scleral buckling, cryopexy

Note: ETDRS, early treatment diabetic retinopathy study; G, gauge; HbA1c, glycosylated hemoglobin; LogMAR, logarithm of the minimal angle of resolution; LP, light perception; NVG, neovascular glaucoma; PRP, panretinal photocoagulation; SD, standard deviation; VEGF, vascular endothelial growth factor; TRD, traction retinal detachment; VH, vitreous hemorrhage

**Table 2:** Structural and functional outcomes

Study	Design	Sample Size	Baseline Perfusion (unit)	Change in Perfusion (unit)	Statistical Significance (*p* value)	Structural Outcomes (RD Rate)	Postoperative Visual Acuity (logMAR)	Choroidal Vascularity Index (CVI)	Choriocapillaris Flow Area (CFA)	Complications	Use of Anti-VEGF	Surgical Techniques Used	Follow-up
Rahimy et al., 2015 [19]	Retrospective	62 eyes of 58 patients	Not specified	Not specified	Not specified	90.3%	Improved from 2.0 ± 0.5 to 1.4 ± 0.8	NA	NA	Early VH (16.1%), delayed VH (4.8%), secondary RRD (17.7%), NVG (8%)	12.9% preoperative bevacizumab	23- or 20-gauge, various tamponade agents (none/air, SO, SF_6_, C_3_F_8_)	6 months
Steinmetz et al., 2002 [20]	Prospective	67 eyes of 62 patients	Not specified	Not specified	Not specified	93% complete reattachment, 100% macular attachment	Improved or stabilized in 51 eyes (72%), 47 eyes (70%) achieving 5/200 or better	NA	NA	VH (37%), RRD (16%), NVG (6%)	Not specified	Multiport Illumination System, 3-port PPV, scleral buckling (24%), gas‒fluid exchange (64%), silicone oil (1%)	12 months
Dikopf et al., 2015 [8]	Retrospective	70 eyes of 55 patients	Not specified	Not specified	Not specified	90% primary reattachment, 99% final anatomical success	Improved from 1.59 (20/800) to 0.68 (20/100),	NA	NA	Elevated postoperative IOP (≥ 22 mm Hg) occurred in 25 eyes, and low IOP (≤ 5 mm Hg) occurred in 2 eyes	70 eyes of 55 patients	Not specified	6 months
Zghal et al., 2019 [22]	Prospective	52 eyes	Not specified	Not specified	Not specified	92.3%	Improved from 1.80 (1/80 vision) to 0.90 (1.25/10 vision), *p* = 0.022p = 0.022p = 0.022	NA	NA	Not specified	Not specified	Transconjunctival 23-gauge vitrectomy	12 months
Russell et al., 2021 [12]	Retrospective	31 eyes of 21 patients	Retinal ischemia present	No appreciable change	Not specified	Resolution of TRD and relief of traction	Improved or stable in individual cases	NA	NA	Residual FVP in some cases, no significant change in retinal ischemia	Some received intravitreal bevacizumab	PPV, various tamponade agents (SF_6_, C_3_F_8_, silicone oil, air)	12 months
Velez-Montoya et al., 2011 [21]	Prospective	12 patients	Not specified	Not specified	Not specified	67% anatomical success at 3 months	Varied; BCVA in Snellen, improvement noted but not statistically significant	NA	NA	Residual FVP, some required additional surgery for reattachment	None preoperative	23-gauge PCL-perfused vitrectomy, silicon oil (5000 cSt) used as tamponade	3 months
Mikhail et al., 2016 [9]	Prospective	109 eyes of 73 patients	Not specified	Not specified	Not specified	91% primary, 98% final anatomical success	Improved from 1.17 (20/300) to 0.812 (20/130), *p* < 0.05p < 0.05p < 0.05	NA	NA	IOP complications: 5 cases hypotony, 11 cases high IOP; 11 eyes with ERM surgery within 1 year; 12 eyes with recurrent VH requiring vitrectomy within 1 year	50% received anti-VEGF	25-G + PPV, various tamponade agents (air, SF_6_, C_3_F_8_, silicone oil), intraoperative laser photocoagulation	12 months
Heimann et al., 1989 [23]	Prospective	106 eyes of 91 patients	Not specified	Not specified	Not specified	64% anatomical success at follow-up, 91 eyes had anatomical success immediately postop	Varied (reported in categories); 35% satisfactory, 13% ambulatory, 51% HM-LP, 14% NLP	NA	NA	Recurrent RDs, residual FVP, elevated IOP in 24 eyes, band-shaped keratopathy in 4 eyes, cataract formation in 40 eyes, rubeosis iridis in 28 eyes (improved in 13, worsened in 3)	Not specified	PPV, silicone oil injection, retinotomy or retinectomy in 32 eyes, ERM surgery, removal of opacified lens in 16 eyes	24 months
Tao et al., 2010 [24]	Prospective	168 eyes of 150 patients	Not specified	Not specified	Not specified	94% reattachment rate	Improved from 2.22 ± 1.22 to 1.24 ± 1.00, *P* < 0.001P < 0.001P < 0.001	NA	NA	7% developed iris neovascularization postoperatively, 6% had persistent RD, NLP in 2 eyes, recurrent RD requiring second surgery in some cases	None used	PPV, removal of ERMs, retinal endolaser coagulation	6 months
Kumar et al., 2014 [25]	Prospective	50 eyes of 50 patients	Not specified	Not specified	*p* < 0.05 for BCVA improvement in both groups	Anatomic attachment achieved in all eyes	23-G: improved from 1.28 ± 0.36 to 0.99 ± 0.38, 25-G: improved from 1.29 ± 0.42 to 0.91 ± 0.55	NA	NA	No postoperative hypotony, iatrogenic breaks in 4 eyes (23-G) and 5 eyes (25-G), intraoperative bleeding in 6 eyes (23-G) and 5 eyes (25-G), ERM in 1 eye per group, cataract formation in 6 eyes (23-G) and 8 eyes (25-G)	Not specified	23-G and 25-G PPV, intraocular tamponade (18% C_3_F_8_ or silicone oil)	12 months
Gurelik et al., 2024 [26]	Prospective	25 eyes of 25 patients	Not specified	Not specified	*p* = 0.154 for visual acuity improvement, *p* = 0.225 for intraocular pressure, *p* = 0.015 for resurgery rate	Retina attached in all patients at the last visit	Group 1: 1.60 ± 0.78, Group 2: 1.56 ± 0.78	NA	NA	No MMC-related complications	Not specified	Vitreoretinal surgery with and without MMC	12 months
Arevalo et al., 2014 [27]	Retrospective	114 eyes of 114 patients	Not specified	Not specified	Not specified	100% (114/114 eyes)	Significant visual improvement (≥ 2 ETDRS lines) in 69.2% (79/114), stable in 22.8% (26/114), decreased (≥ 2 ETDRS lines) in 7% (8/114)	NA	NA	Cataract in 32 (28%) eyes, iatrogenic retinal breaks in 9 (7.8%) eyes, VH requiring another procedure in 7 (6.1%) eyes, phthisis bulbi in 1 (0.9%) eye	Preoperative intravitreal bevacizumab (1.25 mg/0.05 mL)	23-gauge transconjunctival sutureless vitrectomy, EBPD, intraocular tamponade with PFCL and C_3_F_8_ gas	12 months
Stoffelns and Dick, 2000 [28]	Retrospective	84 eyes	Not specified	Not specified	Not specified	73% complete reattachment	85% VA remained unchanged or improved	NA	NA	Rubeosis, secondary glaucoma, repeated VH	Not specified	PPV using membrane peeling and C_2_F_6_-gas	24 months
Qamar, Saleem, and Saleem, 2013 [29]	Prospective	84 eyes	Not specified	Not specified	*p* = 0.010	92.8% complete reattachment	Improved from 2.00 ± 1.24 to 1.24 ± 1.22	NA	NA	None specified	Not specified	PPV without ocular tamponade, endolaser photocoagulation	6 months
Awan et al., 2023 [30]	Prospective	196 eyes	Not specified	Not specified	*p* < 0.001	98% complete reattachment	Improved from 1.86 ± 0.59 to 0.54 ± 0.32	NA	NA	Transient rise in IOP (5.6%), suprachoroidal oil migration, vitreous cavity hemorrhage	Not specified	27-gauge plus PPV, phacoemulsification with lens implantation in 64.3% of cases, endolaser photocoagulation	12 months
Abunajma et al., 2016 [31]	Retrospective	96 eyes	Not specified	Not specified	*p* < 0.001 for multiple factors	90.6% complete reattachment	87.5% had stable vision or at least one line improvement	NA	NA	High IOP, VH, corneal epithelial defect, significant cataract, NVG, severe inflammation, RD	Preoperative intravitreal bevacizumab	PPV, endolaser treatment, intraocular tamponade (silicone oil or gas), cataract extraction surgery	12 months
Wang et al., 2016 [32]	Prospective	66 eyes of 58 patients	Not specified	Not specified	*p* < 0.001 for surgical time and membrane removal time	94.4% primary, 100% ultimate reattachment	Improved significantly in both groups, *p* < 0.001	NA	NA	Transient fibrin formation, transient hypotony, early and late VH, recurrent RD, NVG	Not specified	Four-port bimanual 23-gauge vitrectomy, conventional 23-gauge vitrectomy, phacoemulsification, endolaser photocoagulation, silicone oil or C_3_F_8_ tamponade	12 months
Jain et al., 2019 [33]	Prospective	33 eyes	Not specified	Not specified	Not specified	100% reattachment at 3-month follow-up	Improved from 1.80 to 1.20 logMAR	NA	NA	33% intraoperative iatrogenic breaks, 39.39% needed oil/gas tamponade	15 patients received preoperative intravitreal ranibizumab	PPV with PRH, Constellation system’s proportional reflux feature	3 months
Sokol et al., 2018 [34]	Prospective	90 eyes of 79 patients	Not specified	Not specified	Not specified	Not specified	Not specified	NA	NA	4.4% incidence of MH formation (95% CI, 1.2-11.0%), successful MH closure in 3 out of 4 intervened cases	Not specified	PPV for diabetic TRD	6 months
Sokol et al., 2019 [35]	Retrospective	69 eyes of 61 patients	Not specified	Not specified	*p* < 0.0001	98.6% primary reattachment, 98.6% final attachment	Improved from 1.84 ± 0.61 to 0.93 ± 0.66	NA	NA	VH requiring repeat PPV (7.2%), secondary retinal detachment (1.4%), NVG (1.4%), AHFVP (1.4%), fibrinoid syndrome (1.4%), ERM requiring PPV (1.4%)	Not specified	23-gauge PPV, C_3_F_8_ gas tamponade in 91.3%, silicone oil in 8.7%, combined scleral buckle in 11.6%, combined cataract extraction with intraocular lens implantation in 8.7%, combined pars plana lensectomy in 4.3%	6 months
Falavarjani et al., 2022 [36]	Prospective	38 eyes	Not specified	Not specified	*P* = 0.547 for anatomical success, *P* = 0.840 for BCVA, *P* = 0.007 for surgical facility score	89% (rtPA group), 95% (no injection group)	2.0 (rtPA group), 2.1 (no injection group)	NA	NA	None specified	Not specified	Intravitreal rtPA injection before vitrectomy, 23-gauge vitrectomy	12 months
Duong et al., 2023 [37]	Prospective	79 eyes	Not specified	Not specified	*p* < 0.001 for overall VA improvement	89.9% reattachment	Improved from 1.57 ± 0.72 to 1.08 ± 0.91	NA	NA	VH (50.6%), CME (11.4%), hyphema (14%)	Intraoperative bevacizumab injected in most cases	27-gauge PPV, silicone oil infusion in 6% of cases, endolaser photocoagulation, filtered air, SF_6_, C_3_F_8_ as tamponade agents	12 months
Meier and Wiedemann, 1997 [38]	Retrospective	28 eyes	Not specified	Not specified	Not specified	96% reattachment	50% improved to maximal 0.1	NA	NA	Pallor of optic disk, worsened cataract, recurrent ERM, recurrent TRD, new-onset iris neovascularization, NVG.	Not specified	Modified en bloc excision technique, four-port PPV, silicone oil instillation, fluid‒gas exchange, endolaser coagulation, phacoemulsification, scleral buckle	12 months
Arevalo, 2008 [39]	Retrospective	57 eyes	Not specified	Not specified	Not specified	100% reattachment	Improved (≥ 2 ETDRS lines) in 70.1%, stable in 24.5%, decreased in 5.2%	NA	NA	Phthisis bulbi (1.7%), iatrogenic retinal breaks (7%), VH (7%), cataract (26.3%)	Not specified	EBPD during vitrectomy, PFCL injection, endolaser photocoagulation, air–gas (C_3_F_8_) exchange.	12 months
Rice et al., 1983 [40]	Retrospective	197 eyes	Not specified	Not specified	Not specified	66% reattachment	Improved in 57%, worsened in 35%, unchanged in 9%	NA	NA	Iatrogenic retinal breaks (20%), postoperative VH (23%), RD (43%), NVG (11%), phthisis bulbi (9%)	Not specified	PPV, lens removal, peripheral retinal cryotherapy, scleral buckle, intraocular gas treatment	24 months
La Heij et al., 2004 [41]	Prospective	44 eyes	Not specified	Not specified	*p* = 0.02 for visual acuity improvement	86.3% reattachment	Improved from 20/800 to 20/160	NA	NA	NVG (18%), ischemic optic neuropathy, retinal ischemia, progressive cataract, recurrent TRD, RRD, VH	Not specified	PPV, segmentation, delamination, en bloc excision, wide field viewing systems, PFCLs, silicone oil, multiport illumination system, endolaser, cryocoagulation	12 months
Moon et al., 2015 [42]	Prospective	6 eyes of 4 patients	Not specified	Not specified	Not specified	100% final anatomical success	Functional success in all eyes (Snellen BCVA > 0.1)	NA	NA	Recurrent TRD in one eye, iatrogenic retinal tear in three eyes, severe hemorrhage in one eye	Preoperative bevacizumab (1.25 mg) injected one week prior	Trimanual technique with assistant-controlled light probe illumination, 23-gauge sutureless vitrectomy, noncontact wide-angle viewing system, silicone oil tamponade	3 months
Miller et al., 1980 [43]	Retrospective	43 eyes	Not specified	Not specified	Not specified	65% reattachment	Improved in 26% of eyes	NA	NA	Rubeosis iridis (28%), NVG (16%), VH (49%), phthisis bulbi (14%)	Not specified	PPV, scleral buckling, drainage of subretinal fluid, cryopexy, lentectomy, air injection	12 months
Han et al., 1994 [44]	Prospective	30 eyes	Not specified	Not specified	Not specified	97% reattachment	VA of 5/200 or better in 77%, 20/200 or better in 54%	NA	NA	Iatrogenic retinal breaks (20%), recurrent macular ERM (13%), VH (17%), cataract (30%)	Not specified	Modified en bloc excision technique, PPV, endophotocoagulation, fluid‒gas exchange, scleral buckle, peripheral cryopexy, lensectomy, phacoemulsification with posterior chamber lens insertion	12 months

Note: AHFVP, anterior hyaloidal fibrovascular proliferation; BCVA, best-corrected visual acuity; C_2_F_6_, Hexafluoroethane; C_3_F_8_, octafluoropropane; EBPD, en bloc perfluoro dissection; cSt, centistokes, ERM, epiretinal membrane; ETDRS, early treatment diabetic retinopathy study; FVP, fibrovascular proliferation; G, gauge; IOP, intraocular pressure; HM, hand motion; LogMAR, logarithm of the minimal angle of resolution; LP, light perception; MH, macular hole; MMC, mitomycin C; NA, not applicable; NLP, no light perception; NVG, neovascular glaucoma; PCL, perfluorocarbon liquids; PFCL, perfluorocarbon liquid; PRH, proportional reflux hydrodissection; PPV, pars plana vitrectomy; PRP panretinal photocoagulation; RD, retinal detachment; RRD, rhegmatogenous retinal detachment; rtPA, recombinant tissue plasminogen activator; TRD, tractional retinal detachment; VA, visual acuity; VH, vitreous hemorrhage

### Visual acuity improvement following diabetic vitrectomy

**Fig. 2:**
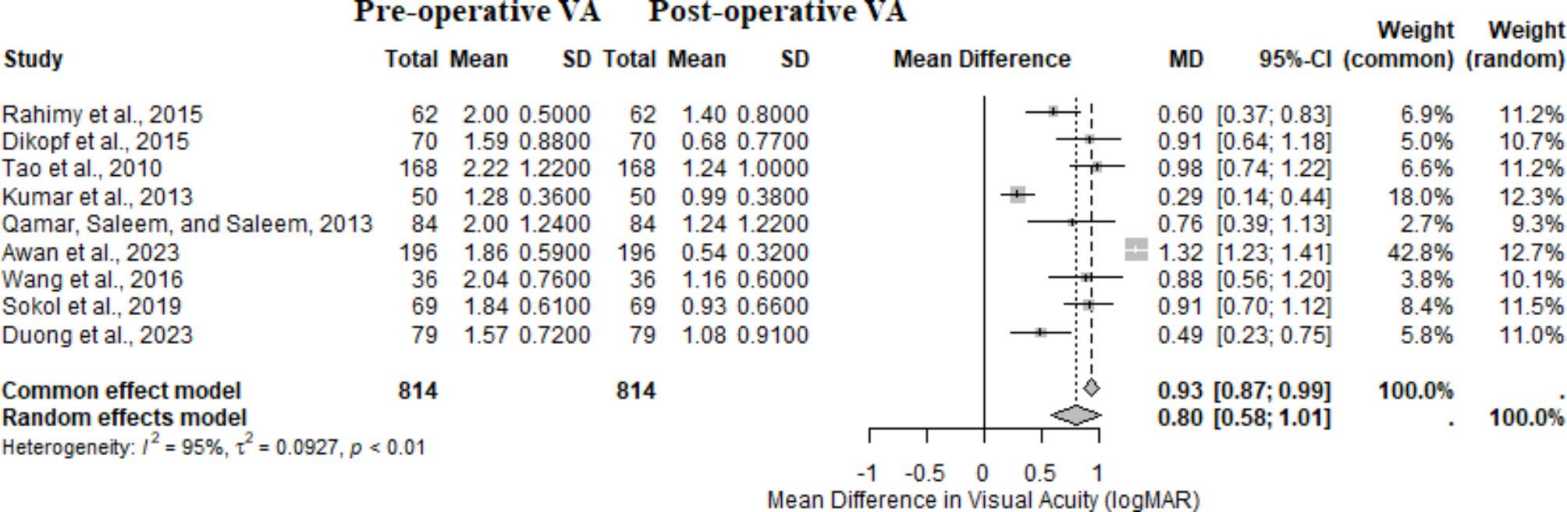
Functional forest plot. A meta-analysis evaluating improvements in visual acuity (logMAR) following diabetic vitrectomy for tractional retinal detachment

The meta-analysis included nine studies with 814 eyes preoperatively and postoperatively treated with diabetic vitrectomy. The analysis revealed a significant visual acuity improvement following surgical intervention for diabetic TRD (Fig. 2). The random effects model indicated a mean difference in visual acuity of 0.80 logMAR (95% CI: 0.58–1.01, *p* < 0.0001), suggesting substantial improvement postoperatively. The individual study results revealed varying degrees of visual acuity improvement. Awan et al. [30] reported the highest mean difference of 1.32 logMAR (95% CI: 1.23–1.41), indicating significant visual gains. In contrast, Kumar et al. (2013) [28] demonstrated the most minor improvement, with an MD of 0.29 logMAR (95% CI: 0.14–0.44). The heterogeneity among studies was high (I² = 95%), indicating variability in the results. This meta-analysis underscores the overall efficacy of diabetic vitrectomy in improving visual outcomes for patients with diabetic TRD despite the observed heterogeneity.

**Fig. 3:**
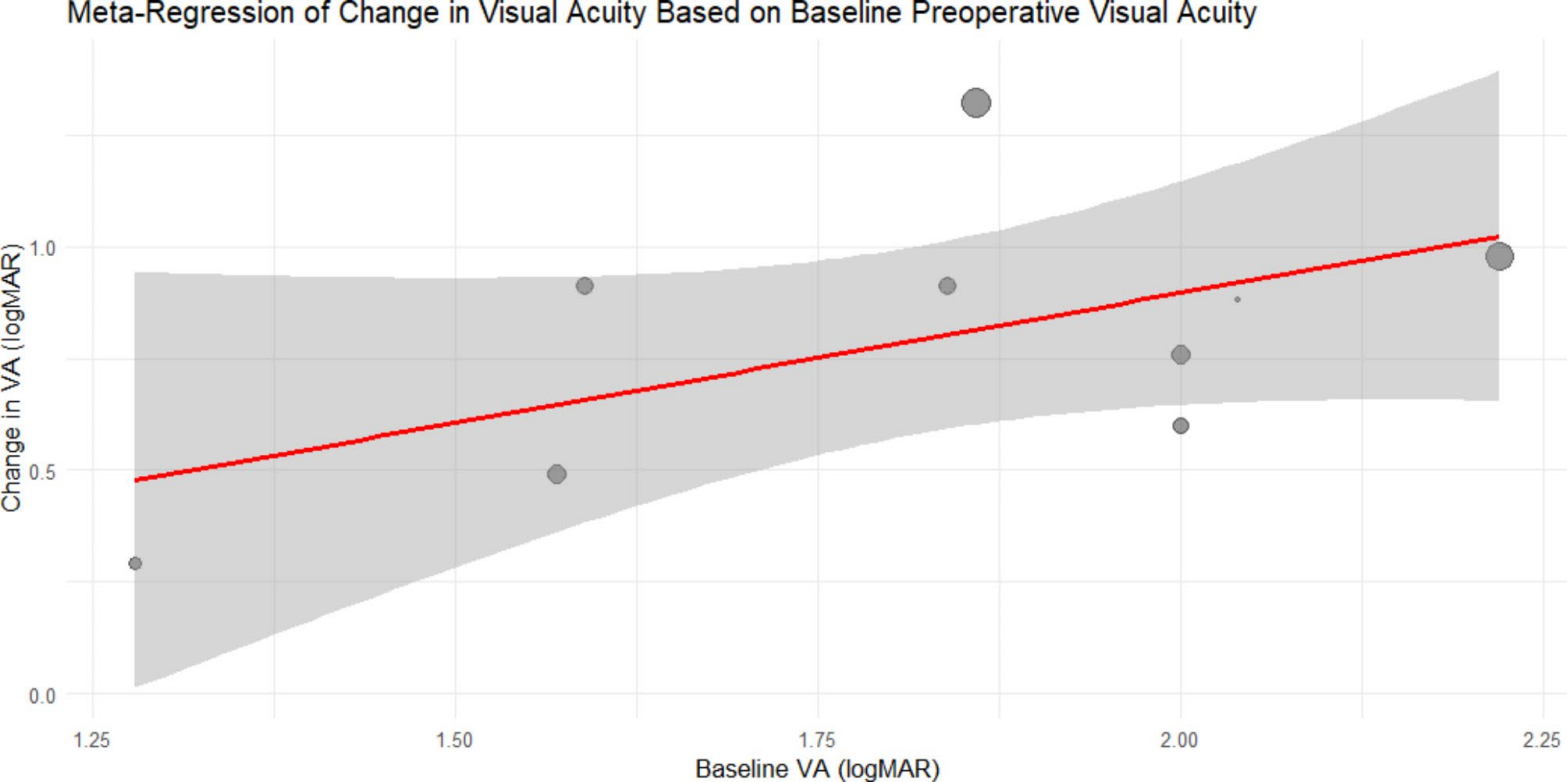
Meta-regression of changes in visual acuity based on baseline preoperative visual acuity. This figure shows the relationship between baseline preoperative visual acuity (logMAR) and changes in visual acuity (logMAR) post-surgery. The red line represents the regression line, and the shaded area indicates the confidence interval. This graph depicts a meta-regression analysis showing a positive relationship between baseline preoperative visual acuity (logMAR) and the change in visual acuity (logMAR) following surgery, with a confidence interval indicated by the shaded area

The meta-regression analysis examined the correlation between preoperative visual acuity (measured in logMAR) and the change in visual acuity (also measured in logMAR) after surgery (Fig. 3).

**Fig. 4:**
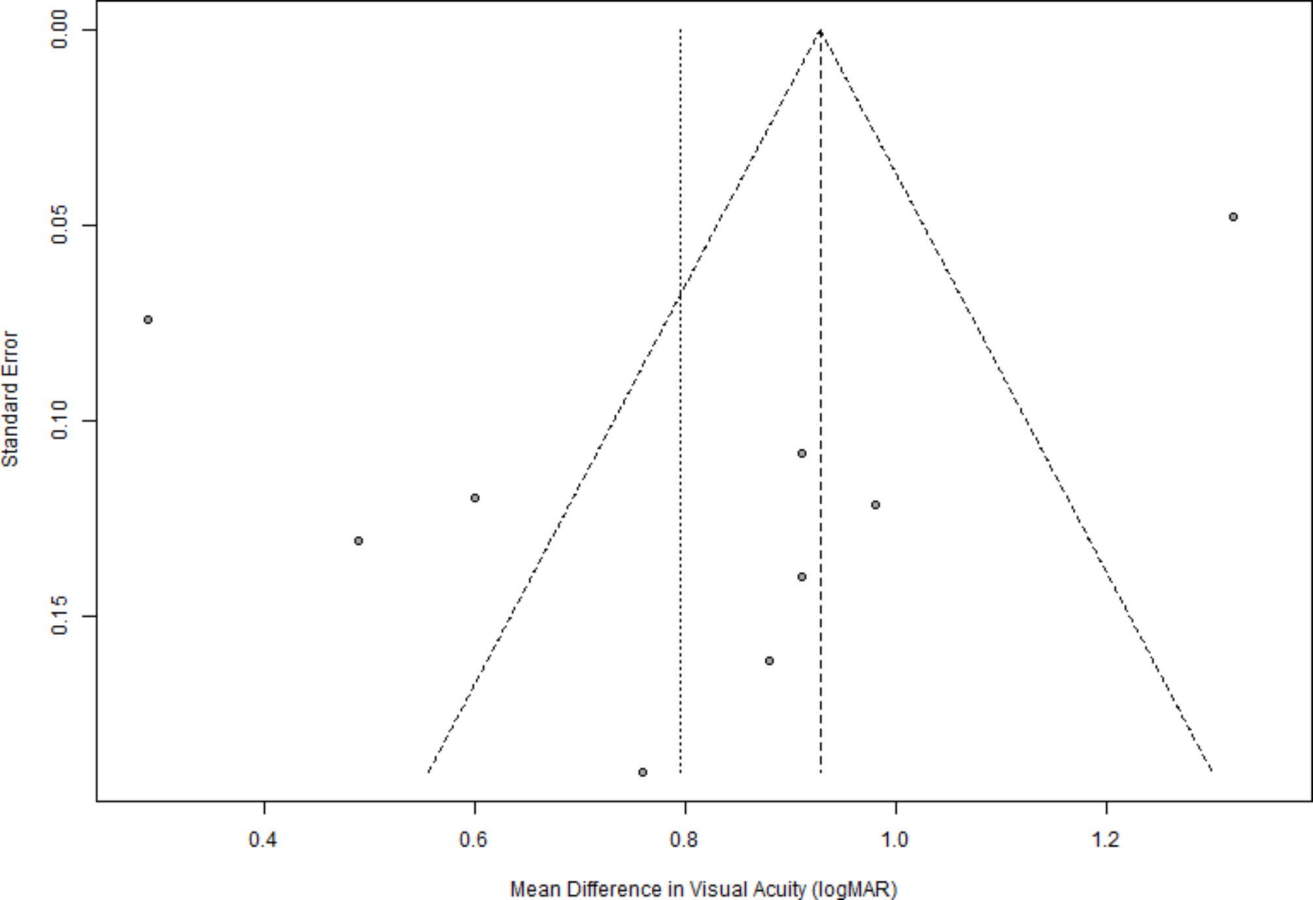
Funnel plot for visual acuity improvement: Funnel plot demonstrating the relationship between the standard error and mean difference in visual acuity (logMAR) following diabetic vitrectomy

The funnel plot (Fig. 4) for the mean difference in visual acuity (logMAR) appeared asymmetrical, indicating potential publication bias. Most studies cluster around the mean difference, with a few showing larger effects and more significant standard errors. This asymmetry suggests that smaller studies with less precise estimates and more significant variability in effect sizes might be overrepresented in the analysis, potentially skewing the results toward more positive outcomes.

### Anatomic success following diabetic vitrectomy

**Fig. 5:**
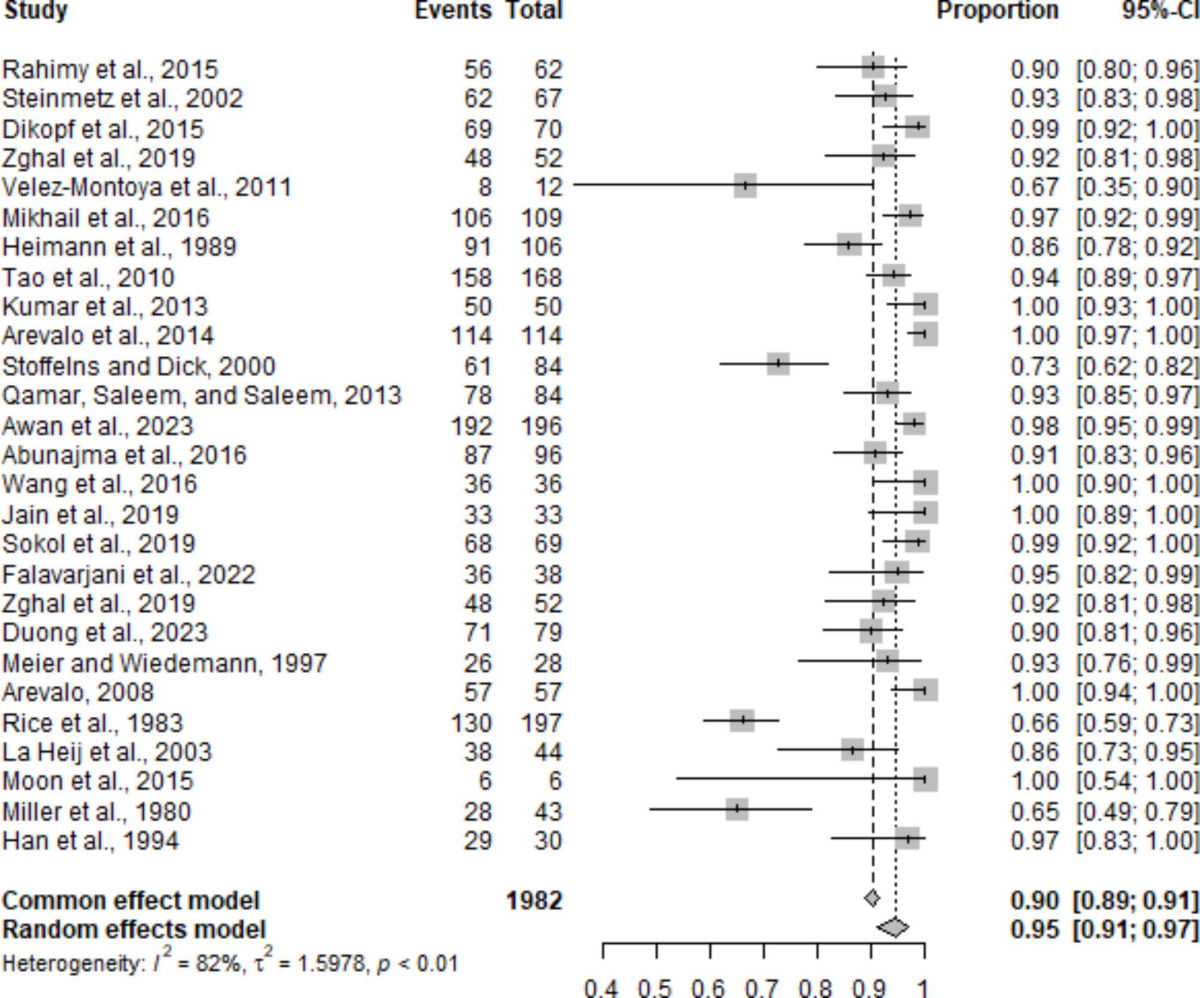
Forest plot showing the pooled retinal reattachment success rates following diabetic vitrectomy for tractional retinal detachment across 27 studies

This meta-analysis assessed the retinal reattachment success rate following diabetic vitrectomy for TRD. A total of 27 studies encompassing 1982 eyes were included in the analysis. The pooled proportion of successful retinal reattachment was high (Fig. 5). The standard effect model yielded a success rate of 90.11% (95% CI: 88.72–91.35%), whereas the random effects model yielded a slightly higher success rate of 94.63% (95% CI: 90.88–96.89%). Individual study success rates varied, with Kumar et al. (2014) [25], Arevalo et al. (2014) [27], Wang et al. (2016) [32], Jain et al. (2019) [33], Moon et al. (2015) [42], and Arevalo (2008) [39] reporting a 100% reattachment rate (95% CI: 92.89–100%).

Heterogeneity among the studies was substantial, with an I² value of 82.2% (95% CI: 75.0–87.3%) and a τ² value of 1.5978, indicating considerable variability in reattachment rates across different studies. This heterogeneity was statistically significant (Q = 146.22, *p* < 0.0001; LRT = 247.04, *p* < 0.0001). The random intercept logistic regression model was employed, using the maximum likelihood estimator for τ² and a logit transformation of proportions. The Clopper‒Pearson confidence interval for individual studies was applied, with a continuity correction of 0.5 in studies with zero cell frequencies, ensuring robust analysis. These findings highlight the effectiveness of diabetic vitrectomy in achieving high rates of retinal reattachment despite the observed heterogeneity among studies.

**Fig. 6:**
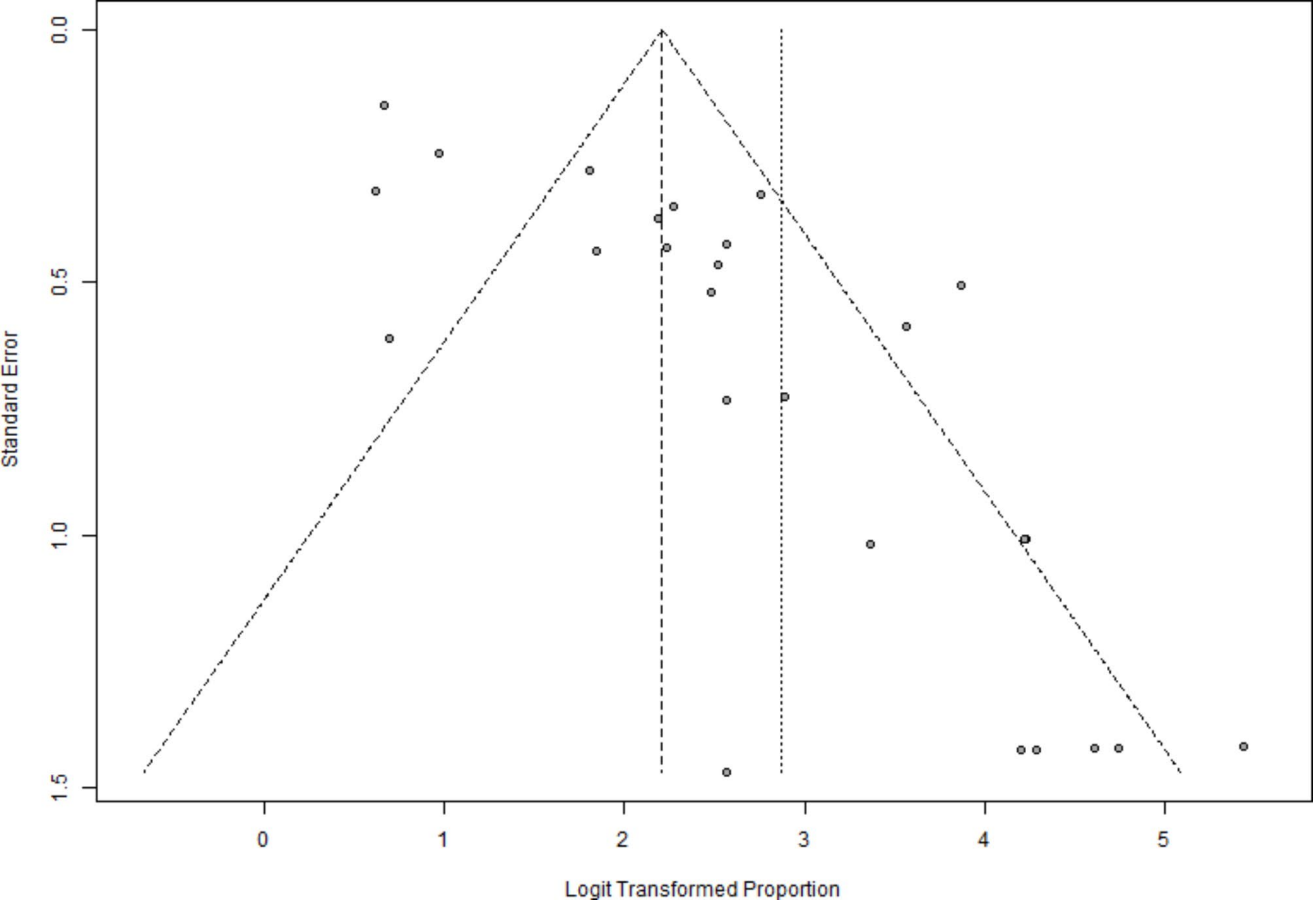
Funnel plot for anatomic success: Funnel plot illustrating the relationship between standard error and logit-transformed proportions of retinal reattachment success rates following diabetic vitrectomy

Like the visual acuity plot, the funnel plot (Fig. 6) also exhibited noticeable asymmetry. The distribution of studies around the logit-transformed mean proportion indicates potential publication bias, with several smaller studies reporting higher success rates and more extensive standard errors. This could imply the selective publication of studies with more favorable outcomes, leading to overestimating the effect size for retinal reattachment success.

### Trim and fill Method and Egger’s regression test

**Fig. 7:**
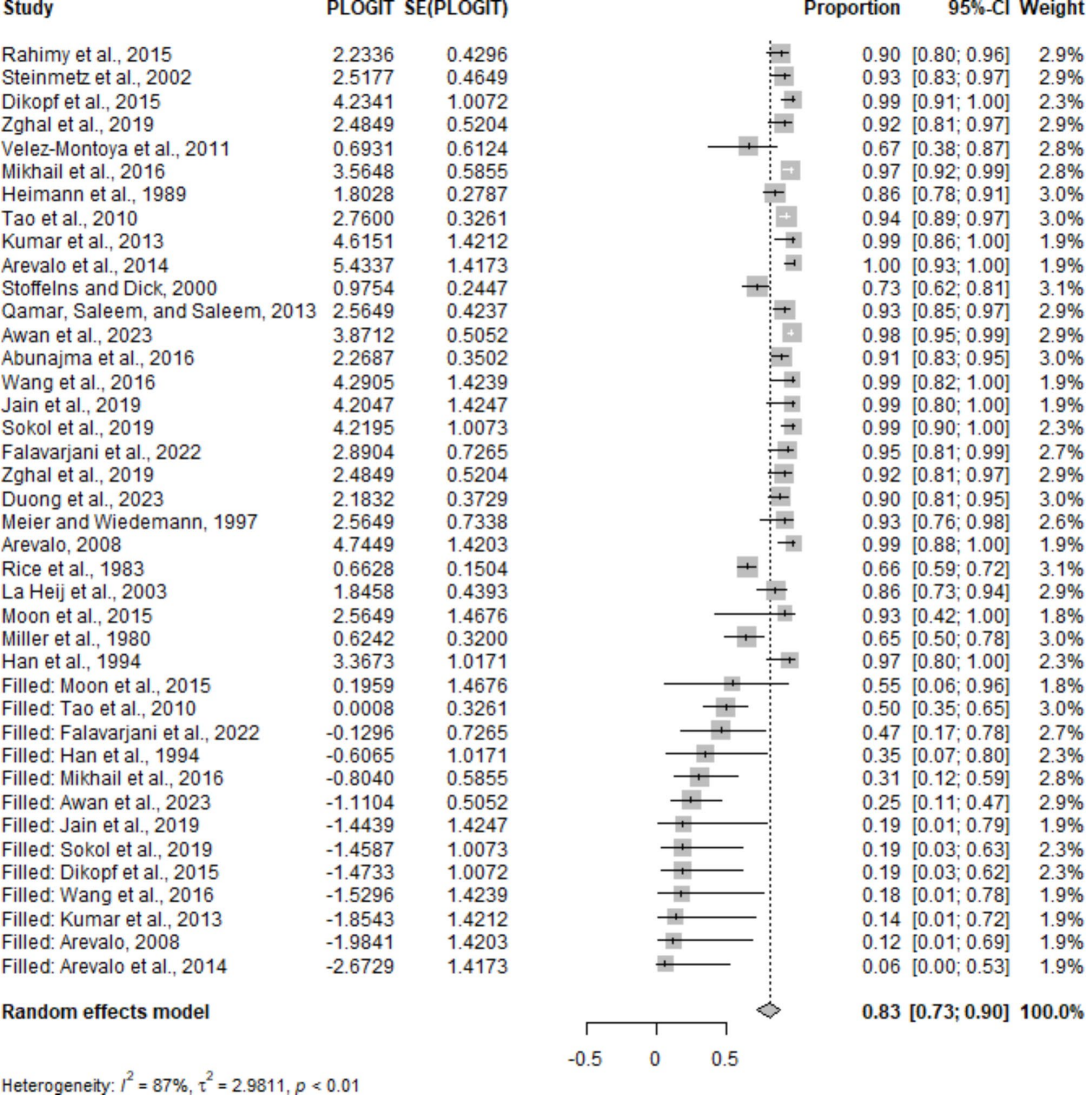
Trim-and-fill method: This figure illustrates the use of the trim-and-fill method to assess publication bias in the meta-analysis of diabetic vitrectomy outcomes

To ensure the reliability of our findings, we conducted a trim-and-fill analysis (Fig. 7) and Egger’s regression test to assess and adjust for potential publication bias in our meta-analyses. For visual acuity improvement, the initial random effects model showed an MD of 0.7959 (95% CI: 0.5809–1.0108), but the trim-and-fill method, which included five hypothetical studies to address funnel plot asymmetry, adjusted this value to an MD of 1.1490 (95% CI: 0.8504–1.4476). Although significant heterogeneity remained (I² = 97.1%), the adjusted model provides a more balanced estimate of visual acuity improvement. However, Egger’s regression intercept test could not be conducted due to an insufficient number of studies (k = 9).

For anatomic success, the initial random effects model reported a pooled proportion of 0.9463 (95% CI: 0.9088–0.9689). The trim-and-fill method adjusted this proportion to 0.8276 (95% CI: 0.7252–0.8972) by adding 13 hypothetical studies. Significant heterogeneity was still present (I² = 86.7%). Egger’s regression test confirmed notable funnel plot asymmetry (t = 6.07, df = 25, p-value < 0.0001), indicating substantial publication bias. These adjustments suggest that our initial findings were influenced by publication bias, particularly for anatomic success, and the adjusted estimates provide a more accurate reflection of the actual effect sizes, reinforcing the reliability of our findings in favor of the effectiveness of vitrectomy in improving visual and anatomic outcomes for diabetic patients, despite the high heterogeneity observed.

**Fig. 8:**
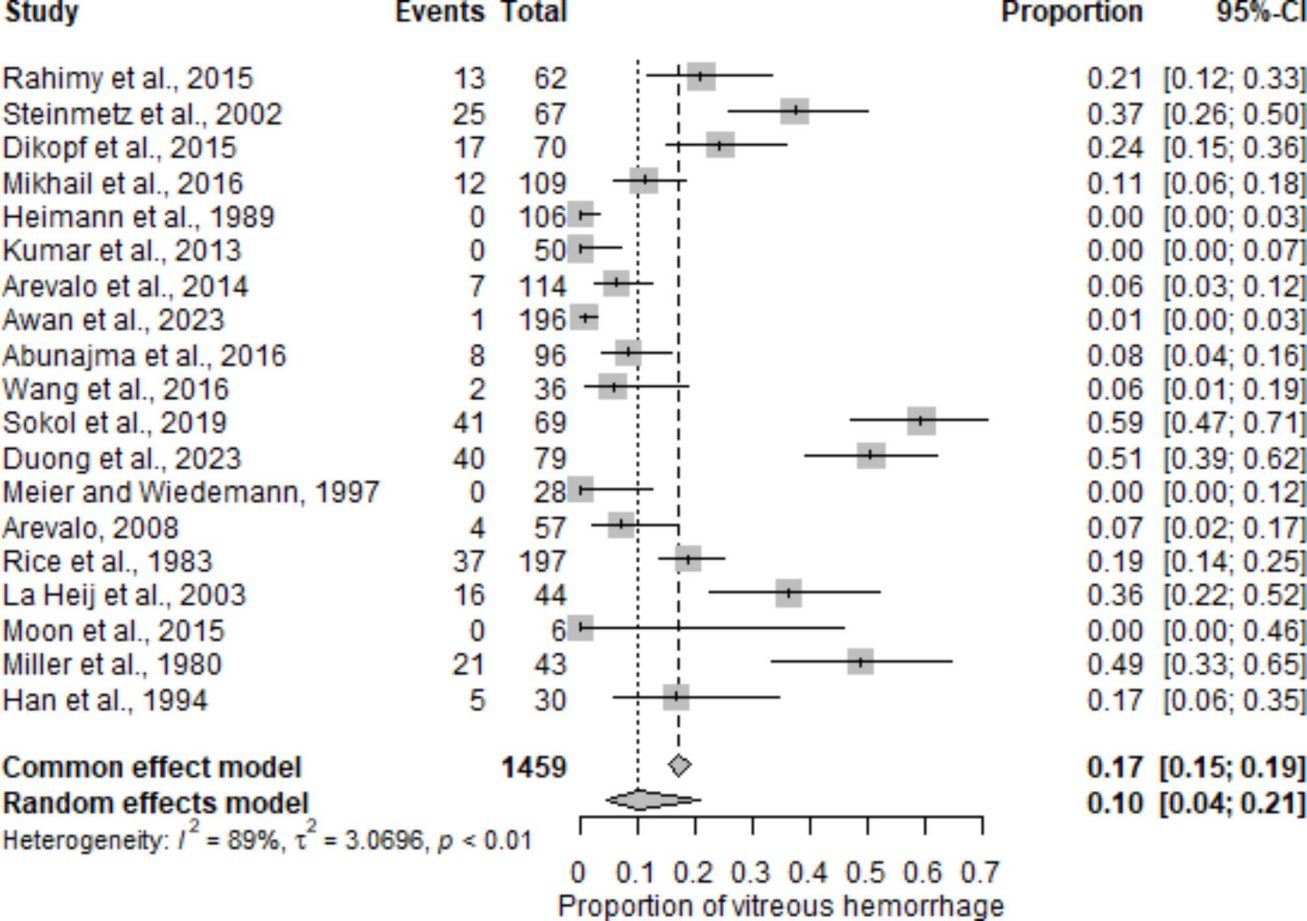
Vitreous hemorrhage: This figure shows the frequency and resolution of vitreous hemorrhage observed postoperatively in patients who underwent diabetic vitrectomy

### Analysis of postoperative complications following diabetic vitrectomy

**Fig. 9:**
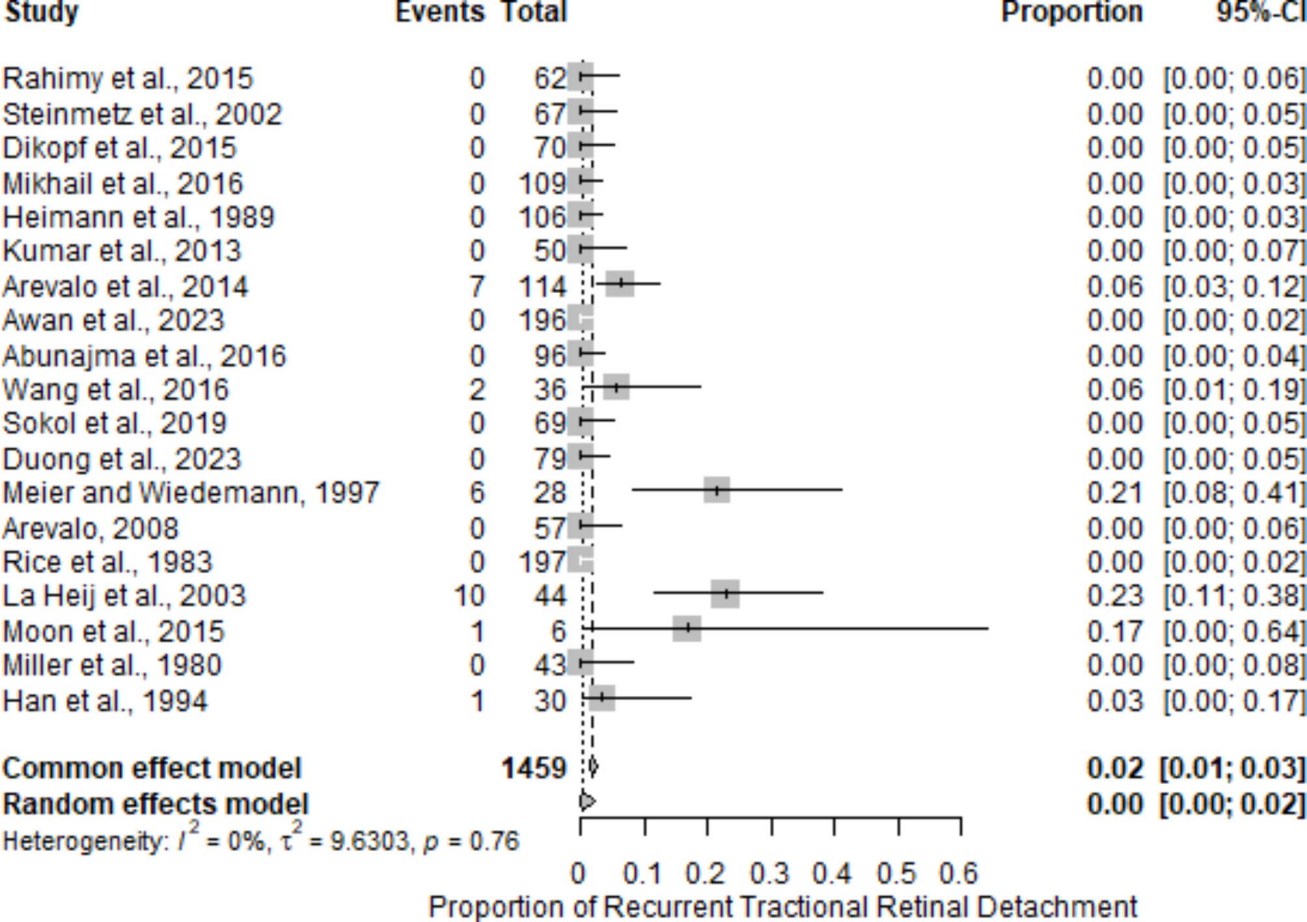
Recurrent tractional retinal detachment: This figure depicts the incidence and characteristics of recurrent retinal detachment following diabetic vitrectomy

The frequency and distribution of postoperative complications following diabetic vitrectomy were systematically analyzed across multiple studies (Table 3). The most reported complication was vitreous hemorrhage (VH), with 19 studies reporting this event, affecting a total of 1459 eyes and a mean postoperative rate of 8.2% (range: 0–41%) (Fig. 8). Epiretinal membrane (ERM) formation was reported in 7 studies involving 500 eyes, with a mean rate of 7.0% (range: 0–17%). Elevated postoperative intraocular pressure (IOP) was noted in 10 studies, encompassing 614 eyes, with an average rate of 7.3% (range: 0–25%). Cataract formation, another significant complication, was documented in 8 studies; it affected 778 eyes and had the highest mean postoperative rate of 10.3% (range: 0–43%). Neovascular glaucoma (NVG) was observed in 4 studies with 247 eyes, for a mean rate of 4.8% (range: 0–8%). Iatrogenic retinal breaks were reported in 6 studies involving 496 eyes, with a mean rate of 5.3% (0–16%). Recurrent TRD was noted in 7 studies involving 470 eyes, with a mean rate of 3.2% (range: 0–10%), as shown in Fig. 9.

**Fig. 10:**
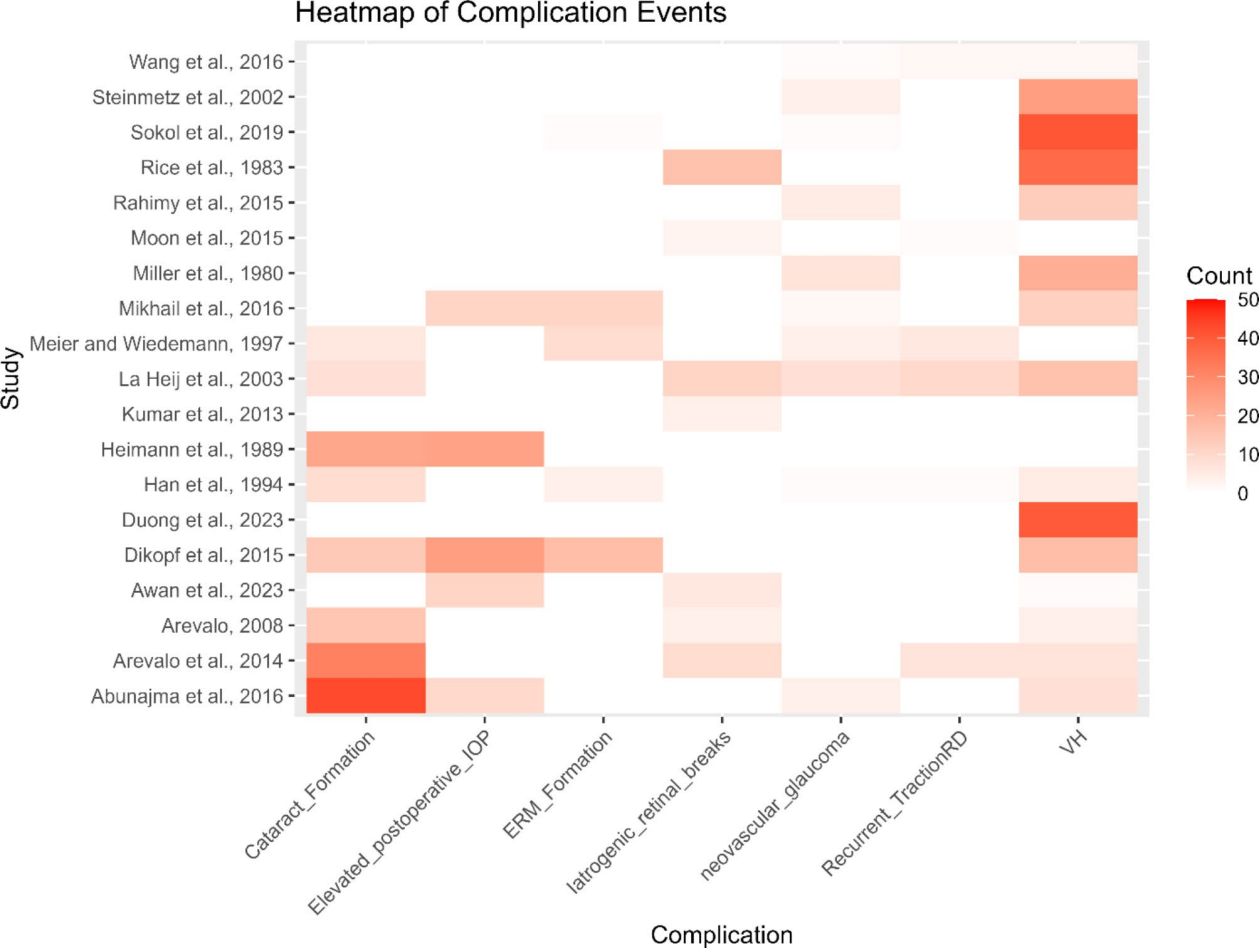
Heatmap illustrating the frequency of postoperative complications across different studies of diabetic vitrectomy

The heatmap (Fig. 10) effectively illustrates the variation in complication rates across different studies, highlighting the most frequently observed complications and the studies with the highest reported rates. This comprehensive analysis highlights the critical need for vigilant postoperative monitoring and management to reduce the occurrence of these common complications in patients undergoing diabetic vitrectomy.

**Table 3:** Postoperative complication frequency

Postoperative Complication	No of studies reporting	Total No of eyes	Mean postoperative rate	Range
VH	19	1459	8.2	0–41
Recurrent TRD	7	470	3.2	0–10
ERM Formation	7	500	7.0	0–17
NVG	4	247	4.8	0–8
Elevated postoperative IOP	10	614	7.3	0–25
Cataract Formation	8	778	10.3	0–43
Iatrogenic Retinal Breaks	6	496	5.3	0–16

Note. IOP, intraocular pressure; ERM, epiretinal membrane; TRD, traction retinal detachment; NVG, neovascular glaucoma; VH, vitreous hemorrhage

### Lack of Comprehensive reporting on choroidal and retinal perfusion markers in Diabetic TRD surgery studies

Despite the critical role that choroidal and retinal perfusion play in the pathophysiology of diabetic retinopathy and its surgical outcomes, many studies reviewed in this systematic analysis did not report essential perfusion markers, such as CVI and CFA. These parameters are pivotal for understanding microvascular changes after surgery and their correlation with visual and structural recovery. The absence of data on the CVI and CFA in most studies limits the ability to assess the effectiveness of vitrectomy fully in restoring ocular perfusion and predicting long-term visual outcomes. This gap underscores the need for future research to incorporate detailed perfusion assessments, including CVI and CFA, alongside traditional structural outcomes such as retinal reattachment rates and visual acuity. Addressing this gap will provide a more holistic understanding of surgical success and help develop targeted interventions to improve patient outcomes in patients with diabetic TRD.

## Discussion

This comprehensive meta-analysis provides substantial evidence of the effectiveness and safety of diabetic vitrectomy for managing TRD. Including 30 studies, meticulously screened as detailed in the PRISMA flowchart, ensures a robust dataset encompassing a diverse patient population. The demographic data, representing 1844 eyes, highlight a varied patient cohort with a mean age of 42–55.3 years and a balanced sex distribution of 511 males and 456 females. The consistent improvement in visual acuity across multiple studies underscores the efficacy of diabetic vitrectomy. The pooled mean difference in visual acuity was significant at 0.80 logMAR (95% CI: 0.58–1.01, *p* < 0.0001) despite high heterogeneity (I² = 95%), indicating substantial postoperative visual gains. However, the observed asymmetry in the funnel plot suggests potential publication bias, indicating an overrepresentation of studies with positive outcomes. The trim-and-fill method further addressed this, which adjusted the mean difference to 1.15 (95% CI: 0.85–1.45), offering a more balanced estimate of the actual effect size.

The significant improvement in visual acuity observed following diabetic vitrectomy is a critical finding of this meta-analysis. The random effects model indicated a mean difference in visual acuity of 0.80 logMAR (95% CI: 0.58–1.01, *p* < 0.0001), demonstrating substantial visual gains for patients undergoing this surgical intervention. The relatively low postoperative visual acuity observed after PPV for diabetic TRD may be attributed to preexisting retinal damage [45], potentially caused by macular ischemia and neurodegeneration, in addition to long-lasting anatomical abnormalities resulting from TRD [46–50]. Our findings support the hypothesis that eyes with better preoperative vision tend to achieve greater postoperative vision. Furthermore, the high heterogeneity (I² = 95%) observed suggests variability in patient populations, surgical techniques, and preoperative conditions. This heterogeneity likely stems from differences in gauge size (e.g., 20-G vs. 23-G/25-G), baseline visual acuity, and adjunctive therapies such as anti-VEGF agents. Inconsistent reporting of baseline characteristics, such as duration of diabetes and systemic comorbidities, further complicates cross-study comparisons. While subgroup analyses for factors like gauge size or preoperative visual acuity could provide insights into these sources of variability, the incomplete reporting of critical variables across studies limits the feasibility of such analyses.

The variability in surgical techniques, such as gauge size (20-G vs. 23-G/25-G/27-G), tamponade agents (e.g., silicone oil, gas), and adjunctive therapies (e.g., anti-VEGF), contributes significantly to the heterogeneity observed in outcomes. Smaller-gauge systems (23-G, 25-G, and 27-G) offer several advantages, including reduced postoperative inflammation, faster recovery times, and fewer suture-related complications [51, 52]. However, their reduced instrument stability and cutting efficiency can limit their use in cases with dense or adherent membranes, such as TRD [51]. According to Shinkai et al. [53], the effectiveness of 27-G vitrectomy for primary retinal detachment, achieving a high success rate of 95.6% for primary reattachment and no significant hypotony-related complications, showcasing the safety of sutureless systems​ [53]. Conversely, 20-G systems are more suited for complex cases due to their larger bore and mechanical stability. Still, they are associated with higher postoperative inflammation [54]. A comparative study by Mohamed et al. [55]. showed that 27-G systems yield superior visual outcomes (mean BCVA improvement to 0.39 ± 0.13 logMAR) at six months compared to 23-G systems (mean BCVA improvement to 0.76 ± 0.37 logMAR), although with longer operative times​ [55].

Additionally, the hybrid use of 23-G and 27-G techniques combines the precision of 27-G with the robustness of 23-G, allowing for shorter surgical times without compromising outcomes​ [54]. Similarly, the tamponade agent choice significantly influences anatomical and functional outcomes. While gas tamponades (e.g., SF_6_, C_3_F_8_) are absorbed spontaneously and offer superior visual acuity outcomes, silicone oil tamponades provide long-term retinal stability but are linked to greater retinal thinning and reduced visual sensitivity [51, 52]. While this meta-analysis did not include sufficient data to conduct a subgroup analysis on these surgical factors, the observed variability highlights the importance of standardizing surgical techniques and reporting outcomes in future studies. Future studies should prioritize prospective, multicenter designs to evaluate these surgical variations’ relative effectiveness systematically.

This remains a significant limitation of the current meta-analysis. Future research should prioritize standardized reporting of surgical techniques, patient characteristics, and outcome measures to reduce heterogeneity and enable more robust comparisons. The trim-and-fill method adjusted the mean difference to 1.15 (95% CI: 0.85 to 1.45), indicating that the initial results may have been influenced by publication bias. As suggested by the asymmetry in the funnel plot, the publication bias remains a significant limitation of this meta-analysis. While the trim-and-fill method adjusted the pooled mean difference in visual acuity improvement from 0.80 logMAR to 1.15 logMAR, it highlights the potential overrepresentation of studies reporting favorable outcomes. This bias may lead to overestimating the benefits of diabetic vitrectomy, particularly for visual acuity improvement and anatomical success rates. Future meta-analyses should mitigate publication bias by incorporating grey literature, unpublished data, and preprints to ensure a more balanced representation of available evidence. Additionally, encouraging the publication of studies with neutral or negative results would enhance the robustness and generalizability of pooled estimates. Despite this adjustment, the high success rates remain consistent, validating the effectiveness of vitrectomy for treating diabetic TRD.

The meta-analysis also revealed a high rate of anatomic success following diabetic vitrectomy, with a pooled retinal reattachment success rate of 90.11% (95% CI: 88.72–91.35%) according to the joint effect model and 94.63% (95% CI: 90.88–96.89%) according to the random effects model. The high success rates reported across 27 studies (encompassing 1982 eyes) underscore the efficacy of vitrectomy in achieving retinal reattachment. This success is critical for preventing further vision loss and maintaining ocular health. A recent meta-analysis and systematic review [56] of PPV for RRD outcomes revealed a primary retinal reattachment rate of 72%, which is significantly lower than the diabetic TRD repair rate presented here. However, the anatomic success rates following multiple surgeries were similar (96%).

The analysis of postoperative complications provides essential insights into the safety profile of diabetic vitrectomy. The most frequently reported complication was VH, observed in 19 studies, with a mean postoperative rate of 8.2% (range: 0–41%) and a cataract formation rate of 10.3%, ranging from 0 to 43%, according to Gupta et al. [57], approximately half of patients underwent surgery for cataracts during the first year after diabetic vitrectomy. Other common complications included ERM formation (7.0%, range: 0–17%), elevated postoperative IOP (7.3%, range: 0–25%), and NVG (4.8%, range: 0–8%). This comprehensive analysis highlights the critical need for diligent postoperative care to mitigate these complications and improve patient outcomes. Despite the robust findings on anatomical and functional outcomes, this meta-analysis was limited by the inconsistent reporting of critical perfusion markers, such as the CVI and CFA. The lack of comprehensive and standardized data on these markers restricted our ability to evaluate postoperative perfusion dynamics and their correlation with functional outcomes. Future studies should prioritize consistently using advanced imaging modalities, such as OCTA, with uniform reporting protocols for perfusion parameters. This would enable more reliable comparisons across studies and deepen our understanding of the perfusion changes following diabetic vitrectomy.

### Strengths and limitations

This meta-analysis has several strengths, including a comprehensive literature search, robust inclusion criteria, and rigorous statistical analyses to address potential biases. Using the trim-and-fill method and Egger’s regression test to assess and adjust for publication bias enhances the reliability of the findings. Additionally, including a large sample size from diverse patient populations strengthens the generalizability of the results. However, there are limitations to consider. The high heterogeneity observed in visual acuity improvement (I² = 95%) and anatomical success (I² = 82.2%) analyses highlights the variability in study methodologies, patient demographics, and surgical techniques. While this heterogeneity provides valuable insights into real-world clinical scenarios, it also limits the precision of pooled estimates.

Furthermore, the publication bias, as indicated by the funnel plot and trim-and-fill analysis, suggests that the reported effect sizes for some outcomes may be overestimated. Another critical limitation is the insufficient reporting of key perfusion markers, such as the CVI and CFA. The need for uniformity in reporting OCT and OCTA outcomes across the included studies posed a significant challenge. While data on perfusion parameters, such as CVI and CFA, were extracted when available, many studies should have reported these metrics in consistent formats or included them, limiting the ability to perform a quantitative synthesis. This inconsistency underscores the need for future studies to adopt standardized imaging protocols and comprehensive reporting frameworks for OCTA data. More precise definitions and consistent measurements of perfusion parameters would enhance the comparability and robustness of future meta-analyses. This gap restricts the ability to assess the impact of vitrectomy on ocular perfusion and its correlation with structural and functional outcomes.

Additionally, the relatively small number of studies included in specific analyses, such as Egger’s test for visual acuity, reduces the statistical power and restricts the ability to draw definitive conclusions. Future research should prioritize the standardization of surgical techniques, including comprehensive perfusion outcomes, and efforts to minimize heterogeneity across studies. Addressing these gaps would enhance the robustness and clinical applicability of future meta-analyses. Despite these limitations, this study provides valuable evidence supporting the efficacy and safety of diabetic vitrectomy, affirming its critical role in managing diabetic retinal complications.

## Conclusion

The current study demonstrates that vitrectomy for diabetic TRD significantly improves visual acuity and achieves high rates of retinal reattachment. Despite considerable heterogeneity among the included studies, the findings suggest that better preoperative visual acuity is related to more favorable postoperative outcomes. However, the lack of comprehensive reporting on critical perfusion markers, such as CVI, CFA, and others, limits our understanding of the full impact of vitrectomy on retinal and choroidal perfusion and its correlation with structural and functional recovery. Additionally, preexisting diabetic choroidal and retinal damage, macular ischemia, and neurodegeneration may restrict postoperative visual recovery. The frequency of postoperative complications, such as elevated postoperative IOP, cataract formation, non-clearing VH, recurrent TRD, NVG, and ERM formation, emphasizes the need for careful postoperative management. Our analysis, adjusted for potential publication bias, supports the overall effectiveness of vitrectomy, highlighting its role in improving visual and anatomical outcomes for patients with diabetic TRD. Future research should prioritize including perfusion markers to enhance our understanding of surgical outcomes.

## Electronic supplementary material

Below is the link to the electronic supplementary material.

Supplementary Material 1

## Data Availability

No datasets were generated or analysed during the current study.

## Abbreviations

BCVA: Best-corrected visual acuity
CFA: Choriocapillaris flow area
CI: Confidence interval
CME: Cystoid macular edema
cSt: Centistokes
CVI: Choroidal vascularity index
C_2_F_6_: Hexafluoroethane
C_3_F_8_: Octafluoropropane, perfluoropropane
DR: Diabetic retinopathy
EBPD: En bloc perfluorodissection
ERM: Epiretinal membrane
ETDRS: The early treatment diabetic retinopathy study
FFA: Fundus fluorescein angiography
FOV: Field of view
HbA1c: Glycosylated hemoglobin
IOP: Intraocular pressure
I^2^: Percentage of variation
τ^2: Logit transformation of proportions
LRT: Likelihood ratio test
LogMAR: Logarithm of the minimal angle of resolution
MD: Mean difference
M/F: Male/female
MH: Macular hole
MIVS: Microincision vitrectomy surgery
NV: Neovascularization
OCT: Optical coherence tomography
OCTA: Optical coherence tomography angiography
PCL: Perfluorocarbon liquid
PICO: Participant, intervention, comparison, outcomes
PFCL: Perfluorocarbon liquid
PRH: Proportional reflux hydrodissection
PRISMA: Preferred reporting items for systemic review and meta-analyses
PPV: Pars plana vitrectomy
PRP: Panretinal photocoagulation
PVD: Posterior vitreous detachment
Q: adjusted p-value
R^2^: Coefficient of determination
RD: Retinal detachment
RRD: Rhegmatogenous retinal detachment
RCTs: Randomized controlled trials
rtPA: Recombinant tissue plasminogen activator
SE: Standard error
SD: Standard deviation
SD-OCT: Spectral domain OCT
SMD: Standardized mean difference
SE: Standard error
SO: Silicon oil
SS-OCTA: Swept-source OCTA
T^**2**^: The difference between the mean values of the two groups. Multivariate probability distribution
T: Regression test
TRD: Traction retinal detachment
SF_6_: Sulfur hexafluoride
RD: Retinal detachment
VEGF: Vascular endothelial growth factor
VA: Visual acuity
VH: Vitreous hemorrhage
