# Supplementary material for: Elucidating postoperative dynamics in tractional retinal detachment: a systematic review and meta-analysis of structural and functional outcomes following diabetic vitrectomy, including an analysis of postoperative complications
Source: BMC Ophthalmol. 2024 Dec 24;24:547. doi: 10.1186/s12886-024-03820-z (PMC11668018; doi:10.1186/s12886-024-03820-z)
Supplement: Supplementary file 1 — Supplementary Material 1 [file 12886_2024_3820_MOESM1_ESM.docx]

**Supplementary information file**

**Elucidating Postoperative Dynamics in Tractional Retinal Detachment: A Systematic Review and Meta-Analysis of Structural and Perfusion Outcomes After Diabetic Vitrectomy.**

**PICO framework:**

**Population:** Patients with tractional retinal detachment due to diabetes

**Intervention**: Diabetic vitrectomy

**Comparison:** Pre-operative vs post-operative conditions after diabetic vitrectomy

**Outcomes:** Structural outcomes (retinal reattachment rates, macular hole closure), functional outcomes (best-corrected visual acuity, etc.), and perfusion outcomes (vessel density, choroidal vascularity index, choriocapillaris flow area, etc.)

| **S.No** | **Keywords** | **Search Results** |
| --- | --- | --- |
| 1 | Tractional Retinal Detachment OR tractional retinal detachment OR TRD OR diabetic retinopathy | 51,416 |
| 2 | Vitrectomy OR Vitrectomies OR diabetic vitrectomy | 24,531 |
| 3 | (1 AND 2) | 4,018 |
| 4 | SD-OCT OR SS-OCT OR spectral-domain optical coherence tomography OR swept-source optical coherence tomography OR retinal reattachment OR macular hole closure OR anatomic success | 40,770 |
| 5 | (3 AND 4) | 676 |
| 6 | OCT-A OR optical coherence tomography angiography OR retinal perfusion OR vessel density OR choroidal vascularity index OR CVI OR choriocapillaris flow area OR CFA | 89,434 |
| 7 | (3 AND 6) | 205 |
| 8 | Postoperative outcomes OR surgical outcomes OR postoperative complications OR complications OR 4 months OR 6 months OR 12 months OR best-corrected visual acuity OR BCVA | 5,816,019 |
| 9 | (5 AND 8) | 616 |
| 10 | (7 AND 8) | 169 |
| 11 | 9 OR 10 | 751 |
| 12 | 11 (Filters Applied: Humans) | 677 |
